# Altered fractional amplitude of low-frequency fluctuation in women with premenstrual syndrome via acupuncture at Sanyinjiao (SP6)

**DOI:** 10.1186/s12991-021-00349-z

**Published:** 2021-05-08

**Authors:** Gaoxiong Duan, Ya Chen, Yong Pang, Zhuo Feng, Hai Liao, Huimei Liu, Zhuocheng Zou, Min Li, Jien Tao, Xin He, Shasha Li, Peng Liu, Demao Deng

**Affiliations:** 1 Department of Radiology, The People’s Hospital of Guangxi Zhuang Autonomous Region, Nanning, 530021 Guangxi China; 2 Department of Radiology, First Affiliated Hospital, Guangxi University of Chinese Medicine, Nanning, 530023 Guangxi China; 3 Department of Acupuncture, First Affiliated Hospital, Guangxi University of Chinese Medicine, Nanning, Guangxi China; 4 Life Science Research Center, School of Life Science and Technology, Xidian University, Xi’an, Shaanxi China

**Keywords:** Acupuncture, Sanyinjiao, FMRI, Fractional amplitude of low-frequency fluctuation, Premenstrual syndrome

## Abstract

**Background:**

Premenstrual syndrome (PMS) is a prevalent gynecological disease and is significantly associated with abnormal neural activity. Acupuncture is an effective treatment on PMS in clinical practice. However, few studies have been performed to investigate whether acupuncture might modulate the abnormal neural activity in patients with PMS. Thereby, the aim of the study was to assess alterations of the brain activity induced by acupuncture stimulation in PMS patients.

**Methods:**

Twenty PMS patients were enrolled in this study. All patients received a 6-min resting-state functional magnetic resonance imaging (rs-fMRI) scan before and after electro-acupuncturing stimulation (EAS) at Sanyinjiao (SP6) acupoint in the late luteal phase of menstrual. Fractional amplitude of low-frequency fluctuation (fALFF) method was applied to examine the EAS-related brain changes in PMS patients.

**Results:**

Compared with pre-EAS at SP6, increased fALFF value in several brain regions induced by SP6, including brainstem, right thalamus, bilateral insula, right paracentral lobule, bilateral cerebellum, meanwhile, decreased fALFF in the left cuneus, right precuneus, left inferior temporal cortex.

**Conclusions:**

Our findings provide imaging evidence to support that SP6-related acupuncture stimulation may modulate the neural activity in patients with PMS. This study may partly interpret the neural mechanisms of acupuncture at SP6 which is used to treat PMS patients in clinical.

*Trial registration*: The study was registered on http://www.chictr.org.cn. The Clinical Trial Registration Number is ChiCTR-OPC-15005918, registry in 29/01/2015.

## Background

Premenstrual syndrome (PMS) is a common gynecological disease and characterized by the cyclic occurrence of emotional and physical symptoms that consistently recur in a cyclic manner during the luteal phase of the menstrual cycle and typically relieve soon after the occurrence of menstruation [1]. About 30–40% of reproductive-age women experience PMS distress, 3–10% of which report severe symptoms round into premenstrual dysphoric disorder (PMDD) [2], which has been classified as depression in newest diagnostic and statistical manual of mental disorders (DSM-V). Pharmacologic intervention for PMS includes hormone therapy and symptomatic treatment, including progesterone, oryzanol, vitamins, and oral antianxiety antidepressants [3]. Even though it works very well, evidence on the efficacy of sustained progesterone and the side effects of antidepressant and anxiolytics limited further application [4].

As an important therapeutic modality in complementary and alternative medicine (CAM), acupuncture could improve microcirculation, balance organ function and adjust mental activities, which has been increasingly and widely accepted by Western countries. Numerous clinical randomized controlled trials have been demonstrated its effectiveness in relieving the physical and psychological symptoms of PMS [5]. Sanyinjiao (SP6, called “gynecological acupoint”), is most commonly acupoint for improving the physical and psychological symptoms in PMS patients [6]. However, lack of research on the mechanism of acupuncture therapy for PMS in existing researches, so it is necessary to study on which.

Functional magnetic resonance imaging (fMRI) has the ability to investigate the functions of human brain and monitor acupuncture-related neural response patterns in humans and provides an opportunity for exploring the neural mechanisms of acupuncture [7]*.* Several fMRI studies on acupuncture have found specific activation patterns in the brain elicited by acupuncture [8, 9]. As a ripe data-driven approach, fractional amplitude of low-frequency fluctuation (fALFF) has been suggested to reflect the intensity of spontaneous activity of whole brain [10]. Our previous study had shown that there were abnormal spontaneous brain activity in PMS patients revealed by fALFF [11], the related results showed that PMS patients had increased fALFF in bilateral precuneus, left hippocampus and left inferior temporal cortex, and decreased fALFF mainly in bilateral anterior cingulate cortex (ACC) and cerebellum at the luteal phase of menstrual. It can be seen that aberrant neural activity of brain in PMS patients might be provided a potential target for acupuncture therapy.

According to our previous study [11], the current study further investigated that whether acupuncture stimulation at SP6 could modulate the abnormal spontaneous brain activity using fMRI combined with fALFF. We hypothesized that down-regulated abnormal neural activity in PMS patients induced by EAS at SP6.

## Methods

### Ethics statement

All procedures of the study were conducted in accordance with the Declaration of Helsinki and permitted by the Medicine Ethics Committee of local Hospital. Each subject was informed about the whole experiment procedure and signed an informed consent.

### Subject recruited

Twenty-three PMS patients were recruited and screened via advertisement in the local university. All of the patients were prospectively screened for 2 months and called for completing a daily record of severity of problems (DRSP) questionnaire decided by Dr. Endicott [12]. Diagnostic criteria for PMS were based on the recommendations and guidelines for PMS [13], while the Diagnostic and Statistical Manual of Mental Disorders-5th Edition (DSM-5) was used to exclude participants with PMDD [14]. All the patients were individually diagnosed by an experienced associated professor gynecologist.

The inclusion criteria for PMS were met: (1) age ranged from 18 to 45 years old, being right-handed; (2) a regular menstrual cycle ranged from 24 to 35 days; (3) the premenstrual symptoms occurred up to 2 weeks before menses in most menstrual cycles; (4) symptoms remitted shortly following onset of menses and were absent during most of the mid-follicular phase of the menstrual cycle; (5) the symptoms were associated with impairment in daily functioning and/or relationships and/or caused suffering, such as emotional, behavioral and physical distress; (6) the menstrual-related cyclicity, occurrence during the late luteal phase of cycle (days − 5 to − 1) and absence during the middle follicular phase (days + 6 to + 10) were documented by repeated observations by the patients based on DRSP, and the mean luteal phase score was at least 30% greater than that of the follicular phase; and (7) the symptoms were not just an exacerbation or worsening of another mental or physical chronic disorders.

The exclusion criteria for patients were as follows: (1) being currently pregnant or lactating; (2) having a history of thyroid disease, dysmenorrhea, gynecological inflammation, menopausal syndrome, hysterectomy or bilateral oophorectomy, mastopathy or cancer, or diabetes or any other structural diseases; (3) having psychiatric disorders by DSM-5 criteria, such as schizoaffective disorder, schizophrenia, organic mental disorder, delusional mental disorder, psychotic features coordinated or uncoordinated with mood or bipolar disorder; (4) treatment with any steroid compound (including oral contraceptives and hormonal intrauterine devices), benzodiazepines, or other psychotropic drugs affecting PMS; (5) having any MRI contraindications; and (6) smoking or alcohol abuse.

### Experimental paradigm

This study adopted the non-repeated event-related (NRER) paradigm designed by Qin et al. [15]. Every participant underwent two 6-min fMRI scans, which included a 6-min resting-state scan before and after EAS. Acupuncture manipulation was completed by an experienced and licensed acupuncturist (device type: HuaTuo-brand, SDZ-V-type, Shanghai, China). EAS was executed by inserting a stainless-steel disposable needle (specifications: 0.30 mm × 45 mm; Huatuo brand, Suzhou, Jiangsu, China) into the left leg at acupoint SP6. Another electrode was connected to the acupuncture needle, which was superficially inserted into a point 1.0 cm away from SP6. Due to the physical characteristics of women and sex hormone levels, the test date was arranged during the late luteal phase of the menstrual cycle. All of the tests were performed between 20:00 and 22:00 to ensure a relatively stable and low level of endogenous cortisol and estradiol [16]. Each subject was informed to keep their eyes closed, but to stay awake during the fMRI scan. After the fMRI scan, all subjects were asked to recall Deqi sensations, which are thought to have therapeutic effects in clinical practice, and to complete the visual analog scale (VAS) to assess *Deqi* sensations, includes sensations of soreness, numbness, fullness, heaviness, tingling, coolness, warmth, sharp pain, dull pain, aching and pressure [17].

### fMRI data acquisition

MRI data were acquired using a 3.0-T Siemens Magnetom Verio MRI System (Siemens Medical, Erlangen, Germany) at the Department of Radiology, First Affiliated Hospital, Guangxi University of Chinese Medicine, Nanning, Guangxi, China. To avoid head movement, each subject’s head was immobilized by foam pads in a standard eight-channel birdcage head coil. FMRI images were acquired with a single-shot gradient-recalled echo planar imaging (EPI) sequence with the parameters as following: repetition time (TR)/echo time (TE) = 2000 ms/30 ms, flip angle = 90°, field of view (FOV) = 240 mm × 240 mm, matrix size = 64 × 64, slice thickness = 5 mm and slices = 31. High-resolution T1-weighted images were then obtained with a volumetric three-dimensional spoiled gradient recall sequence with the parameters as following: TR/TE = 1900 ms/2.22 ms, FOV = 250 mm × 250 mm, matrix size: 250 × 250, flip angle = 9°, slice thickness = 1 mm and 176 slices.

### Image preprocessing

Preprocessing was performed with SPM8 (SPM8).The first 10 volumes of each functional time series were removed to avoid the instability of the initial MRI signal. The remaining images were corrected for acquisition time delay between different slices and realigned to the first volume. The head motion parameters were calculated by estimating the translation in every direction and the angular rotation on each axis for every volume. If the translation was more than 1.5 mm in any cardinal direction and the rotation was more than 1.5° in each of the orthogonal *x*, *y* and *z* axes, the subject was discarded. The realigned functional images were then spatially normalized to the Montreal neurological institute space using the normalization parameters estimated by T1 structural image unified segmentation, re-sampled to 3 mm × 3 mm × 3 mm voxels. Several sources of spurious variance, such as the estimated motion parameters, average blood oxygenation level-dependent (BOLD) signals in ventricular and white matter regions, were dislodged from the images. After removing the variance, linear drift was removed and temporal filter (0.01–0.08 Hz) was then performed on the time series of each voxel to reduce the effect of low-frequency drifts and high-frequency noise.

### fALFF analyses

The fALFF analyses were performed using the DPARSF software as the description from Zou et al. study [10]. The preprocessed images were temporally band-pass filtered (0.01 < *f* < 0.08 Hz) to reduce low-frequency drift and high-frequency respiratory and cardiac noise. For each voxel, the preprocessed time series were transformed into the frequency domain using a fast Fourier transform (FFT) without band-pass filtering. The square root was calculated at each frequency of the power spectrum. The sum of the amplitude across 0.01–0.08 Hz was divided by that of the entire frequency range (0–0.25 Hz).

### Statistical analyses

Paired t-tests were used to measure patterns of neural activity (fALFF maps) in PMS patients before and after acupuncture at SP6. The contrast threshold was set at *p* < 0.05 (false discovery rate [FDR] corrected) and cluster size > 30.

## Results

### Demographic and clinical results

**Fig. 1:**
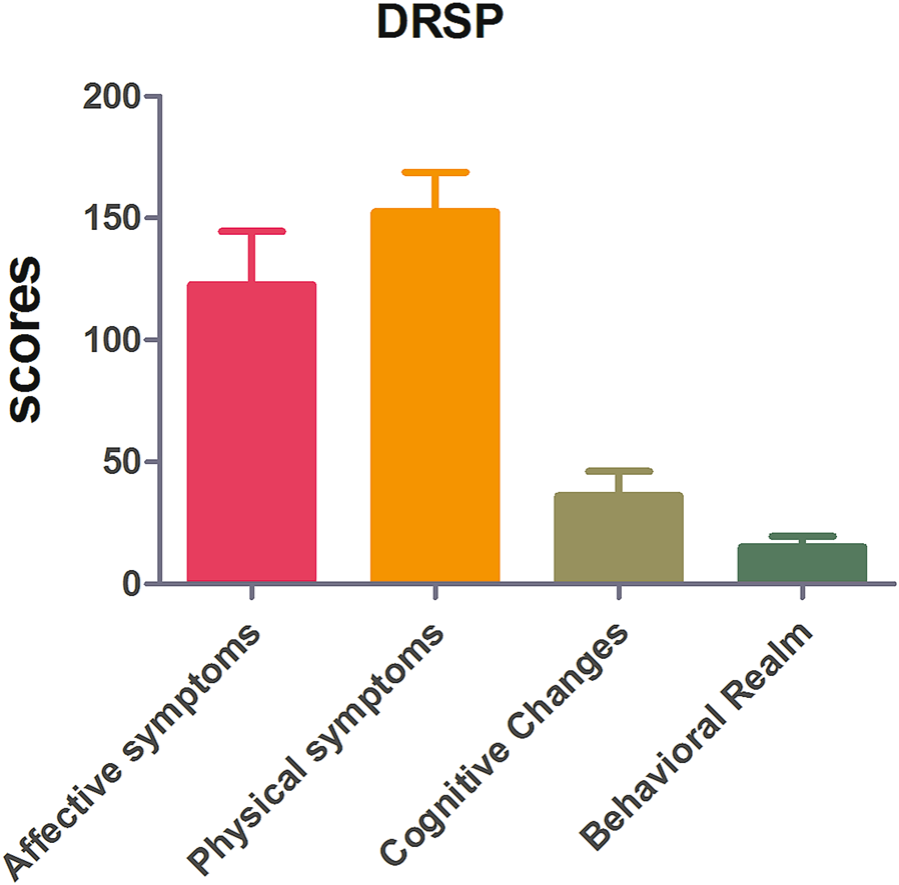
DRSP score features in PMS. We subdivided the DRSP evaluation entries into four symptoms to better understand the clinical characteristics of PMS

Due to obvious head motion, three participants were excluded. Twenty PMS patients were included in the final analysis. We subdivided the DRSP evaluation entries into four symptoms to better understand the clinical characteristics of PMS. The detailed results are shown in Table 1 and Fig. 1.

**Table 1:** Demographic and clinical characteristics for the study

Variable	PMS
Number	20
Age (years)	21.85 ± 1.72
BMI	18.60 ± 1.71
Menophania (years)	13.75 ± 1.44
Length of menstrual cycle (days)	29.95 ± 1.76
Menstruation (days)	5.60 ± 1.09

All values are mean ± standard deviation (SD)

*PMS* premenstrual syndrome, *BMI* body mass index

### Acupuncture sensation results

**Fig. 2:**
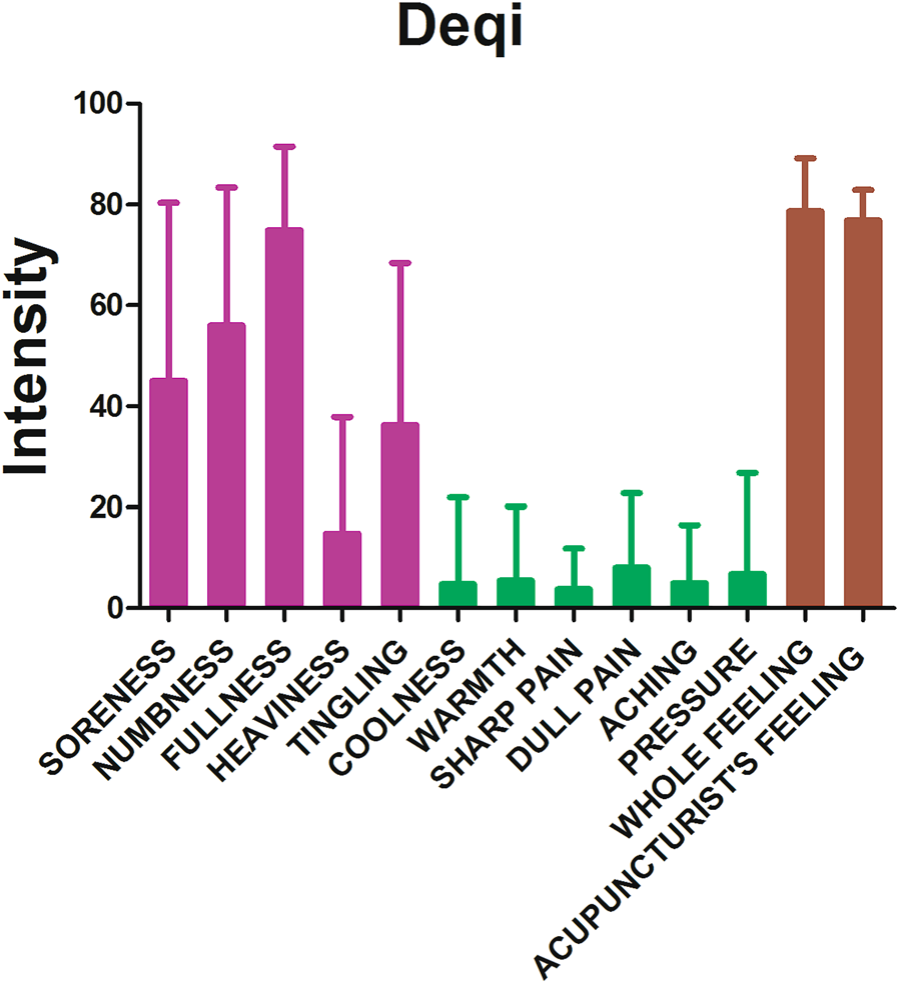
Results of psychophysical analysis. The soreness, numbness, fullness, heaviness and tingling were the primary Deqi sensations. The error bar stood for standard deviation (SD) of the Deqi sensations

The prevalence of Deqi sensations reported by patients was expressed as frequency and intensity (Fig. 2). The current results showed that main Deqi sensations included fullness, dull pain, numbness, soreness, tingling and heaviness.

### Imaging results

**Fig. 3:**
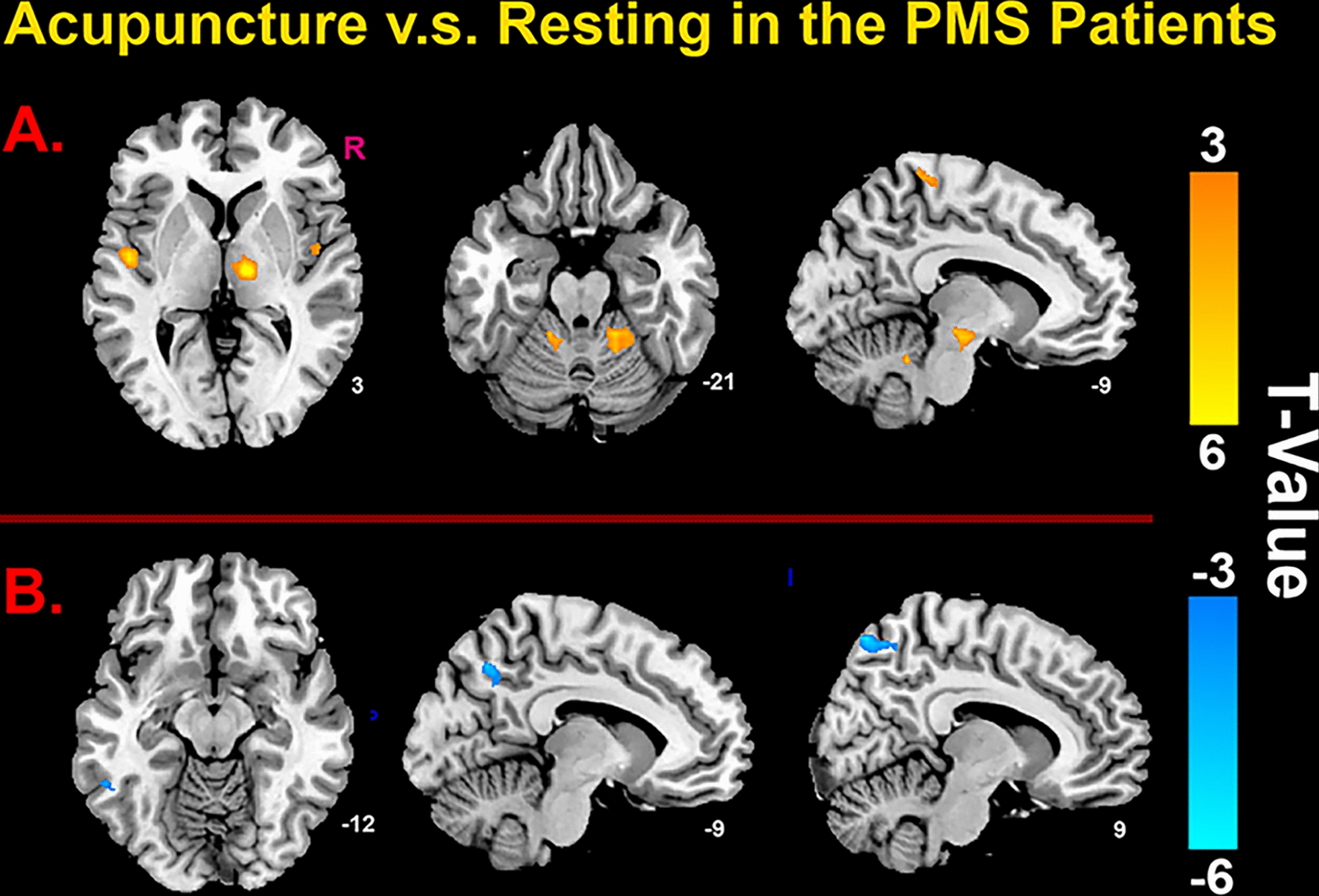
Results of HIPP FC in subjects. Upper image indicated that differences of the HIPP FC between patients and healthy subjects; lower image showed that differences of abnormal HIPP FC in patients before and after EAS at Baihui (GV20)

Compared with pre-EAS at SP6, increased fALFF in several brain regions induced by SP6, including brainstem, right thalamus, bilateral insula, right paracentral lobule, bilateral cerebellum, and decreased fALFF in the left cuneus, right precuneus, left inferior temporal cortex (Fig. 3; Table 2).

**Table 2:** Main localization of brain maps by comparing electro-acupuncture stimulation (EAS) with resting state in premenstrual syndrome (PMS) patients

Regions	BA	MNI			*T*-value	Vol.
		*X*	*Y*	*Z*		
R_Paracentral_Lobule	4	3	− 42	72	4.93	55
L_Insula	48	− 42	− 9	3	4.73	51
R_Insula	48	46	− 3	3	4.17	38
R_Thalamus		12	− 15	3	6.83	76
Brainstem		− 6	− 21	− 9	4.59	28
L_Cerebellum		− 18	− 63	6	4.90	48
R_Cerebellum		6	− 69	9	5.73	91
L_Cuneus	31	− 18	− 60	24	− 4.00	42
R_Precuneus	31	12	− 63	24	− 5.13	55
L_inferior temporal cortex	20	− 54	− 48	− 9	− 3.86	24

## Discussion

Using fMRI technology combined with fALFF data-processing approach, our previous study found aberrant spontaneous brain activity in PMS patients compared with healthy controls, the related results showed that PMS patients had increased fALFF in bilateral precuneus, left hippocampus and left ITC, and decreased fALFF mainly in bilateral ACC and cerebellum at luteal phase [11]*.* The current study further investigated that whether acupuncture stimulation at SP6 could modulate the abnormal spontaneous brain activity in PMS patients during the luteal phase of menstrual. The results indicated that PMS patients had decreased fALFF in right precuneus, left ITC and left cuneus, and increased fALFF mainly in bilateral cerebellum, bilateral insula, brainstem, right thalamus and right paracentral lobule. Our findings might serve as a possible interpretation of the mechanisms of acupuncture at SP6 modulation to the abnormal neural activity in PMS patients.

### Down-regulated aberrant brain regions in PMS

Precuneus was known to be one of the most important regions related to emotion, cognition, and memory, and involved in mental disorder [18, 19]. Emotional and cognition symptoms were commonly happened in PMS during the luteal phase. Our previous found increased fALFF of bilateral precuneus in patients with PMS, but also significant positive correlations between the fALFF in the precuneus and DRSP scores [11]. Interestingly, decreased fALFF of right precuneus were found in PMS patients induced by EAS at SP6. We inferred that EAS at SP6 could down-regulate the activity of precuneus in PMS patients during the luteal phase of menstrual, which might be the therapeutic mechanism of acupuncture for PMS.

Moreover, Halbreich pointed out that PMS might be related to abnormal temporo-limbic circuitry which might be heredity or acquired in a very early age, and the abnormal temporo-limbic circuitry referred to the etiology of PMS [1]. Previous studies suggested that PMS patients showed significantly increased reactivity and functional connectivity in the ITC during luteal phase, that is in line with our outcomes [11]. In the present study, PMS patients indicated decreased fALFF in left ITC induced by EAS at SP6. We speculated that acupuncture was able to regulate the activity of ITC in PMS patients, which might be another therapeutic mechanism of acupuncture for PMS.

As it is known to us, the fundamental function of cerebellum refers to maintaining postural balance and coordinating voluntary movement. Furthermore, study has exhibited that cerebellum is implied in many functions, such as cognitive and affective [20, 21]. As the severe form of PMS, PMDD was related to cerebellum, the literature suggested that cerebellum contributed to a wide range of negative mood and behaviors involving emotion, pain, and executive functions in female with PMDD [22]. Our previous findings showed decreased fALFF in bilateral cerebellum in patients with PMS [11], and the present study found increased fALFF in bilateral cerebellum induced by EAS at SP6. We considered that acupuncture could modulate the activity of cerebellum in PMS patients, which might be therapeutic mechanism of acupuncture for PMS.

### Affected brain function of PMS

The brainstem is an important center for processing pain information via the spinothalamic tract. The thalamus is a key relay station for the transmission of nociceptive information to the cerebral cortex, which may hold the key to pain consciousness [23]. The brainstem and thalamus reticular formation are important centers for processing pain information and integrating pain sensation, which is the third level of pain information transmission. The insula encodes both the intensity and the laterality of painful and non-painful thermal stimuli, but may also have a role in affective pain processing [24–26]. The paracentral lobule located in the upper medial part of the precentral gyrus and the postcentral gyrus, was composed of primary motor cortex (MI) and the primary somatosensory cortex (SI) according to the somatotopic map [27]. These brain regions mentioned above were significantly related to the process of pain perception. The current study showed increased fALFF in brainstem, right thalamus, bilateral insula and right paracentral lobule in patients with PMS induced by EAS at SP6. Our findings were similar to the ones from several previous studies of pain processing pathway (i.e., from brainstem to thalamus to insula/limbic system). We speculated that EAS at SP6 could affect the state of pain-related network in PMS patients, which might be a therapeutic mechanism of acupuncture for PMS.

As a vital region of the visual network, the cuneus was within the extrastriate cortex and is involved in visual selective attention by relaying top-down information from the attention network to visual areas [28]. Aberrant activity in the cuneus was found in migraineurs during the spontaneous headache and after pain relief [29]. For the decreased fALFF in cuneus in PMS patients elicited by SP6 in our study, we inferred that EAS at SP6 might affect the function of visual network. However, such findings need further study to verify.

There are several limitations in this study. Firstly, the present study only demonstrated increased/decreased fALFF values in the brain of PMS patients regulated by acupuncture at SP6, but did not show that these fALFF values were “PMS specific” as there were no healthy controls or sham conditions. In the future, the use of a control condition would be advised. Secondly, although our study indicated that there was a trend between Deqi sensations and fALFF changes of certain brain regions, we were unable to extract any meaningful results from these complicated correlations. Finally, our sample size of PMS patients was not very big; therefore, the present findings should be retested with a larger sample size in the future.

## Conclusions

In summary, according to our previous study [11], the current study further investigated that whether acupuncture stimulation at SP6 could modulate the abnormal spontaneous brain activity in PMS patients during the luteal phase of menstrual using fMRI technology combined with fALFF data-processing approach. Results indicated that aberrant brain regions of PMS could be down-regulate induced by EAS at SP6, which including precuneus, ITC and cerebellum. Furthermore, regulated the pain-related network (brainstem, insula, thalamus and paracentral lobule) and visual network (cuneus) in PMS patients elicited by SP6. Our findings provide neuroimaging evidence to better understand the underlying mechanisms of acupuncture at SP6 in PMS patients.

## Data Availability

The raw data supporting the conclusions of this manuscript will be made available by the authors, without undue reservation, to any qualified researcher.
